# Prevalence of social risk factors and social needs in a Medicaid Accountable Care Organization (ACO)

**DOI:** 10.1186/s12913-022-08721-9

**Published:** 2022-11-19

**Authors:** Katherine H. Schiavoni, Kristy Helscel, Christine Vogeli, Anne N. Thorndike, Rebecca E. Cash, Carlos A. Camargo, Margaret E. Samuels-Kalow

**Affiliations:** 1 Mass General Brigham, Population Health Management, 299 Revolution Drive, Somerville, MA 02145 USA; 2 Departments of Medicine and Pediatrics, Massachusetts General Hospital, Boston, MA USA; 3 Harvard Medical School, Boston, MA USA; 4 The Mongan Institute, Massachusetts General Hospital, Boston, MA USA; 5 Department of Medicine, Massachusetts General Hospital, Boston, MA USA; 6 Department of Emergency Medicine, Massachusetts General Hospital, Boston, MA USA

**Keywords:** Health-related social needs, Population health management, Health surveys, Primary health care, Cross-sectional studies

## Abstract

**Background:**

Health-related social needs (HRSN) are associated with higher chronic disease prevalence and healthcare utilization. Health systems increasingly screen for HRSN during routine care. In this study, we compare the differential prevalence of social risk factors and social needs in a Medicaid Accountable Care Organization (ACO) and identify the patient and practice characteristics associated with reporting social needs in a different domain from social risks.

**Methods:**

Cross-sectional study of patient responses to HRSN screening February 2019-February 2020. HRSN screening occurred as part of routine primary care and assessed social risk factors in eight domains and social needs by requesting resources in these domains. Participants included adult and pediatric patients from 114 primary care practices. We measured patient-reported social risk factors and social needs from the HRSN screening, and performed multivariable regression to evaluate patient and practice characteristics associated with reporting social needs and concordance to social risks. Covariates included patient age, sex, race, ethnicity, language, and practice proportion of patients with Medicaid and/or Limited English Proficiency (LEP).

**Results:**

Twenty-seven thousand four hundred thirteen individuals completed 30,703 screenings, including 15,205 (55.5%) caregivers of pediatric patients. Among completed screenings, 13,692 (44.6%) were positive for ≥ 1 social risk factor and 2,944 (9.6%) for ≥ 3 risks; 5,861 (19.1%) were positive for social needs and 4,848 (35.4%) for both. Notably, 1,013 (6.0%) were negative for social risks but positive for social needs. Patients who did not identify as non-Hispanic White or were in higher proportion LEP or Medicaid practices were more likely to report social needs, with or without social risks. Patients who were non-Hispanic Black, Hispanic, preferred non-English languages or were in higher LEP or Medicaid practices were more likely to report social needs without accompanying social risks.

**Conclusions:**

Half of Medicaid ACO patients screened for HRSN reported social risk factors or social needs, with incomplete overlap between groups. Screening for both social risks and social needs can identify more individuals with HRSN and increase opportunities to mitigate negative health outcomes.

**Supplementary Information:**

The online version contains supplementary material available at 10.1186/s12913-022-08721-9.

## Background

Health-related social needs (HRSN) are associated with high chronic disease prevalence, poor disease control [1–4], and high health care utilization in both adults and children [5–7]. Increasingly, health systems are screening patients for HRSN during routine care and integrating responses into the electronic medical record (EMR), with the goal to refer or provide resources to address identified needs [8, 9]. Screening for HRSN has also become a priority for public payors in the Accountable Care Organization (ACO) model as a strategy to prevent and treat chronic disease [10–12], and several states allow use of Medicaid funds to directly address HRSN like food and housing [13, 14].

Screening for HRSN may be completed using many instruments, which can include questions on social risk factors as well as social needs [15–17]. The relationship between social risk factor and social need screening is unclear. Consistent with prior literature, we use the term *health-related social needs* to mean a group of individual-level adverse social determinants of health, such as those assessed in a screening instrument [10]. We use *social risk factors* or *social risks* as the specific adverse social and economic conditions associated with poor health as measured on the individual level, for example food insecurity. We use the term *social needs* when individuals express their own preferences and priorities to address these conditions, such as requesting assistance with food [18, 19].

Previous studies have shown that the identification of social risk factors is not always consistent between different screening instruments assessing the same domain, such as housing risk [20]. There has also been substantial variability in the extent to which individuals identified as having social risk factors on screening instruments report social needs by requesting additional assistance [21–24]. In smaller studies of patient questionnaires in a research context, 8.6 to 26% of participants who screened negative or declined to answer social risk screening questions still indicated they were interested in receiving resources for social needs [23, 25, 26]. It is therefore not clear how social risk factor and social need screening overlap in identifying HRSN in a population.

In our study, we sought to understand the prevalence of social risk factors and social needs in a large population screened for HRSN as part of routine clinical care. We included both patient and practice level characteristics in our model, drawing from the Drivers of Health framework, which includes indirect factors (such as public policy, gender, and racial identity) that affect direct factors (such as environment, access to and quality of healthcare, and social circumstances) that affect health outcomes [27].

The goals of this study were: (1) to compare the differential prevalence of social risk factors and social needs in a Medicaid ACO population, specifically describing the characteristics of patients who would be missed by screening for social risk factors only, and (2) to identify the patient and practice characteristics associated with reporting social needs in a different screening domain from social risk factors.

## Methods

### Study design and setting

We conducted a cross-sectional study of patient responses to a HRSN screening questionnaire from February 2019 to February 2020. This period was chosen to reflect full implementation of the HRSN screening program after launch in March 2018, but before the disruption in routine care that occurred due to the COVID-19 pandemic.

We examined HRSN screening responses from 114 outpatient primary care practices across a large Medicaid ACO in an integrated health system in Massachusetts including those in academic medical centers, community physician-hospital organizations, and affiliated physician groups. Practices were located in urban, suburban, and rural settings. Of included practices, 15 (10.4%) were in Community Health Center locations, 73 (64.0%) were owned by the health system, and 41 (36.0%) were private practices affiliated with the health system. Included practices actively screened during the entire study period and had ≥ 5 patient responses.

Patients eligible for the study sample were enrolled in Massachusetts Medicaid (MassHealth); were in the Medicaid ACO for at least 11/13 months during the study period; and completed the questionnaire either during a primary care visit or by phone with staff at an included practice. Of the approximately 107,900 individuals in the Medicaid ACO in 2019, 31,156 were eligible for inclusion. Patients may have completed the screening more than once if they had multiple qualifying primary care encounters. All completed items on the screening questionnaire were analyzed and incomplete screening items were treated as missing completely at random.

### Health-related social needs screening

HRSN screening was conducted as part of routine primary care for patients in the Medicaid ACO beginning in 2018. The questionnaire was available in English or Spanish for patients to complete through an online portal, on a tablet before primary care visits, or verbally with staff assistance, with the goal to complete annually. Patient responses were imported into the EMR. For patients 15 years or younger, a parent or caregiver completed the questionnaire on their behalf.

The screening questionnaire assessed social risk factors in eight domains (food, housing, medication, transportation, utilities, family care, employment, education), as well as social need as defined by a request for more information in any of the same eight domains (Supplemental Fig. 1). We used request for more information to define social need in this study because answers expressed patient prioritization of that domain and preference for additional engagement. The questionnaire was created for institutional use by compiling portions of publicly available validated screening tools and adding additional questions for domain completeness. Prior to implementation, the institutional questionnaire was tested with patient focus groups and modified as needed. While the tool in its entirety was not formally validated, we used it in this study to understand the results of pragmatically implemented HRSN screening in a real-world setting.

### Outcome and predictor variables

Outcome variables included (1) reporting social needs among those who screened positive or negative for social risks, and (2) reporting social needs in a concordant or discordant domain as the social risk factor. Concordant domain was defined as reporting social need in any domain where a patient also screened positive for a social risk factor. Discordant domain was defined as reporting social need in a domain where a patient screened negative for social risk, while screening positive for a different social risk factor.

Predictor variables on the patient level included pediatric age (≤ 18 years), sex, race, ethnicity, language, and whether ≥ 3 social risk factors were positive. Patient-level information was obtained from the EMR. At the practice level, predictor variables included proportion of patients with Limited English proficiency (LEP) and with Medicaid insurance where payor data was available. Practice-level information was obtained from aggregated EMR data of patients attributed to the practice by having an insurance-assigned primary care provider or ≥ 3 practice visits. Twenty-five practices located at two participating academic medical centers had payor composition data available (referred to as the “payor subset”) and were examined in a secondary analysis. Datasets were linked using a patient medical record number, date of questionnaire completion, and EMR location.

### Analytic approach

We used a log binominal multivariable regression model with generalized estimating equations (GEE) to understand the patient and practice characteristics associated with social need, and with reporting social need in a concordant or discordant domain as social risk. We estimated prevalence ratios using a binomial distribution, log link function, and working independence correlation structure. We chose a GEE model to address potential non-independence of the observations and a hierarchical model to account for clustering of patients at the practice level.

After evaluating the model suitability of continuous variables, we found practice proportion LEP and Medicaid failed the assumption of linearity. We also found high correlation (*ρ* = 0.79) between the continuous practice LEP and Medicaid variables, with concern for collinearity in the model. Therefore, we included both practice-level LEP and Medicaid composition as categorical variables using quartiles and combined the categorical variables into a single indicator of high-need practice environment defined as top quartile for both LEP and Medicaid (2 social factors), top quartile for 1 social factor, or no top quartile ranking.

We conducted statistical analyses using SAS 9.4 software. A two-sided p ≤ 0.05 defined statistical significance. This study follows RECORD and STROBE reporting guidelines for observational studies of routinely-collected health data [28]. It was approved by the Institutional Review Board at Mass General Brigham.

## Results

### Study population

The study population included 27,413 patients at 114 primary care practices who completed a HRSN screening questionnaire during the February 2019 to February 2020 study period (Table 1). The mean patient age was 24.2 years, with 55.5% of the population age 18 years or younger. Less than half of the population identified as non-Hispanic White and 19.2% preferred a language other than English for medical care.

**Table 1:** Characteristics of Medicaid Accountable Care Organization (ACO) patients who completed health-related social needs (HRSN) screening

	n (Total = 27,413)	%
Age		
Pediatric (0–18 years)	15,205	55.5%
Adult (≥ 19 years)	12,208	44.5%
Sex		
Male	15,663	57.1%
Female	11,750	42.9%
Race / Ethnicity		
Hispanic or Latino	9599	31.3%
Non-Hispanic Black or African American	3126	10.2%
Non-Hispanic other race	2291	7.5%
Non-Hispanic White	13,354	43.5%
Non-Hispanic unavailable race	2333	7.6%
Primary Language		
English	21,483	78.4%
Spanish	4094	14.9%
Other	1167	4.3%
Declined	669	2.4%

The full sample of 114 primary care practices included 54 adult medicine, 27 family medicine or medicine-pediatrics, and 33 pediatric practices. There was a median 3.5 full time equivalent (FTE) providers per practice (range 0.5–15.2, IQR 3.75) with a median 6,907 attributed patients per practice (range 438–27,528, IQR 5,850). Practices had median 2.8% patients with LEP (range 0.1–56.2%, IQR 7.0%).

### Screening response

The 27,314 patients in the study sample completed 30,703 HRSN screening questionnaires (Table 2), representing 87.7% (27,314/31,156) of eligible patients and 25.3% (27,314/107,900) of the ACO. Of completed screenings, 13,736 (44.6%) were positive for ≥ 1 social risk factor and 2,954 (9.6%) for ≥ 3 risks. The most prevalent domains of social risk were education (21.2%), food insecurity (16.7%), unemployment (11.6%), and difficulty paying for utilities (10.1%).

**Table 2:** Social risk factors (positive screening response) and social needs (request for more information) among health-related social needs (HRSN) questionnaires completed

Social Risk Factors						
	n (Total = 30,703)			%		
Domains Positive for Risk						
0	17,011			55.4%		
1	7386			24.0%		
2	3380			11.0%		
3 +	2944			9.6%		
Positive Domain						
Food insecurity	5113			16.7%		
Housing insecurity	2376			7.7%		
Medication affordability	1106			3.6%		
Transportation	1848			6.0%		
Utilities	3111			10.1%		
Child or family care	1303			4.2%		
Employment	3549			11.6%		
Education	6518			21.2%		
Social Needs						
	All HRSN Screens		With Social Risk Factors		Without Social Risk Factors	
	n	%	n	%	n	%
Any request	5861	19.1%	4848	35.4%	1013	6.0%
Food insecurity	1390	4.5%	1227	9.0%	163	1.0%
Housing insecurity	1904	6.2%	1662	12.1%	252	1.5%
Medication affordability	492	1.6%	431	3.2%	61	0.4%
Transportation	977	3.2%	848	6.2%	129	0.8%
Utilities	1845	6.0%	1614	11.8%	231	1.4%
Childcare	1045	3.4%	857	6.3%	188	1.1%
Care for elder or disabled	487	1.6%	399	2.9%	88	0.5%
Job search or training	1311	4.3%	1141	8.3%	170	1.0%
Education	1566	5.1%	1375	10.0%	191	1.1%

Among completed screenings, 5,861 (19.1%) reported a social need, including 4,848 (35.4%) positive for social risk factors and 1,013 (6.0%) negative for social risk factors (Table 2). Patients who screened positive for risk in any domain most often reported social need in unstable housing (12.1%), difficulty paying for utilities (11.8%), education (10.0%), and food insecurity (9.0%). Notably, patients who screened negative for risks in all domains still reported social needs most often in housing (1.5%), utilities (1.4%), education (1.1%), and childcare (1.1%).

Patients who screened positive for social risk factors in any domain were significantly more likely to report social needs if they were female, identified as a race/ethnicity other than non-Hispanic White, preferred Spanish or another non-English language, or received care at a practice in a higher quartile of patients with LEP (Table 3). Those who screened positive for 3 or more social risks were also significantly more likely to report social needs.

**Table 3:** Patient and practice characteristics associated with expressing social needs, with and without social risk factors on health-related social needs (HRSN) screening

	Social Needs with Social Risks Factors			Social Needs without Social Risk Factors		
	PR	CI	*P*-value	PR	CI	*P*-value
Age						
Adult (≥ 19 years)	–	–	–	–	–	–
Pediatric (0–18 years)	0.9	0.9–1.0	0.03	0.8	0.7–1.0	0.02
Sex						
Male	–	–	–	–	–	–
Female	1.1	1.0–1.1	0.03	1.0	0.9–1.2	0.53
Race/ethnicity						
NH White	–	–	–	–	–	–
NH Black	1.4	1.4–1.5	< 0.001	2.1	1.7–2.6	< 0.001
Hispanic	1.3	1.2–1.4	< 0.001	1.8	1.5–2.3	< 0.001
NH other race	1.2	1.2–1.3	< 0.001	1.5	1.2–2.0	0.001
Primary language						
English	–	–	–	–	–	–
Spanish	1.1	1.1–1.2	< 0.001	1.2	0.9–1.5	0.19
Other language	1.1	1.0–1.2	0.03	1.4	1.0–1.9	0.08
Number of social risks						
< 3 social risks	–	–	–	–	–	–
≥ 3 social risks	2.1	2.0–2.2	< 0.001	–	–	–
Practice level LEP						
Quartile 1 practice LEP	–	–	–	–	–	–
Quartile 2 practice LEP	1.2	1.1–1.4	0.001	1.1	0.8–1.4	0.68
Quartile 3 practice LEP	1.3	1.2–1.5	< 0.001	1.5	1.2–2.0	0.002
Quartile 4 practice LEP	1.4	1.3–1.6	< 0.001	1.4	1.2–1.8	0.002

*Abbreviations*: *CI* Confidence interval, *LEP* Limited English proficiency, *NH* Non-Hispanic, *PR* Prevalence ratio

### Social need without social risk factors

Among those who screened negative for social risk factors, patients who identified as a race/ethnicity other than non-Hispanic White or who received care at practices in the top quartiles of patients with LEP were significantly more likely to report social needs (Table 3). Patients who preferred languages other than English were not more likely to report social need when they did not have social risk factors. With or without social risk factors, caregivers of pediatric patients were significantly less likely to report social needs.

### Social need discordant to social risk factors

In the full study population, patients who identified as non-Hispanic Black, preferred a language other than English, or received care at a practice in the top two quartiles of patients with LEP were significantly more likely to report social need in a domain different from their social risk factor (Table 4).

**Table 4:** Patient and practice characteristics associated with expressing social need in domains concordant and discordant with social risk factors on health-related social needs (HRSN) screening

	Social Need in Concordant Domain			Social Need in Discordant Domain		
	PR	CI	*P*-value	PR	CI	*P*-value
Age						
Adult (≥ 19 years)	–	–	–	–	–	–
Pediatric (0–18 years)	0.9	0.9–1.0	0.02	1.0	0.9–1.1	0.66
Sex						
Male	–	–	–	–	–	–
Female	1.1	1.0–1.2	0.01	1.0	0.9–1.1	0.64
Race/ethnicity						
NH White	–	–	–	–	–	–
NH Black	1.5	1.4–1.6	< 0.001	1.4	1.2–1.6	< 0.001
Hispanic	1.3	1.2–1.4	< 0.001	1.2	1.0–1.4	0.09
NH other race	1.3	1.2–1.4	< 0.001	1.2	0.9–1.4	0.17
Primary language						
English	–	–	–	–	–	–
Spanish	1.0	1.0–1.1	0.40	1.6	1.3–1.9	< 0.001
Other language	1.0	0.9–1.1	0.97	1.5	1.2–1.8	0.001
Number of social risks						
< 3 social risks	–	–	–	–	–	–
≥ 3 social risks	3.0	2.8–3.1	< 0.001	0.6	0.5–0.7	< 0.001
Practice level LEP						
Quartile 1 practice LEP	–	–	–	–	–	–
Quartile 2 practice LEP	1.2	1.1–1.4	0.002	1.3	1.0–1.7	0.09
Quartile 3 practice LEP	1.3	1.2–1.5	< 0.001	1.3	1.0–1.7	0.04
Quartile 4 practice LEP	1.4	1.3–1.6	< 0.001	1.4	1.1–1.8	0.006

*Abbreviations*: *CI* Confidence interval, *LEP* Limited English proficiency, *NH* Non-Hispanic, *PR* Prevalence ratio

#### Payor subset secondary analysis

We also analyzed a subset of 11,093 patients at 25 practices where payor composition data was available (Supplemental Tables 1– 4). Demographic data for this subset are provided in Supplemental Table 1. Compared to the larger sample, slightly more patients in this group reported social risk factors (46% versus 44.6%) and social needs (23.0% versus 19.1%) (Supplemental Table 2).

Among this subset of 25 practices, 14 were adult medicine, 5 were family medicine or medicine-pediatrics and 6 were pediatric practices. Practice size included median 4.5 FTEs (range 0.5–15.2, IQR 2.5) and 7,779 attributed patients (range 2,404–27,528, IQR 4,302). Practices had median 11.7% patients with LEP (range 1.1–56.3%, IQR 27.1%). Practice payor composition included median 13.9% Medicaid (range 2.8–54.9%, IQR 30.7%); 14.9% Medicare (range 0.0–39.1%, IQR 15.9%), and 61.7% commercial payors (range 30.3–83.4%, IQR 21.4%).

##### Social need without social risk factors

In the payor subset, patients who identified as non-Hispanic Black, spoke a language other than English or Spanish, or received care in a highest quartile practice for both LEP and Medicaid were significantly more likely to report social needs when they did not report social risk factors (Supplemental Table 3). In this smaller group, Hispanic identity and Spanish language preference were no longer significantly associated with reporting social needs.

##### Social need discordant to social risk factors

Consistent with the larger dataset, non-Hispanic Black identity, non-English language preference continued to predict reporting domain discordant social needs, along with receiving care in the highest-need practice environment with top quartile proportion of patients with LEP and Medicaid enrollment (Supplemental Table 4).

## Discussion

**Fig. 1:**
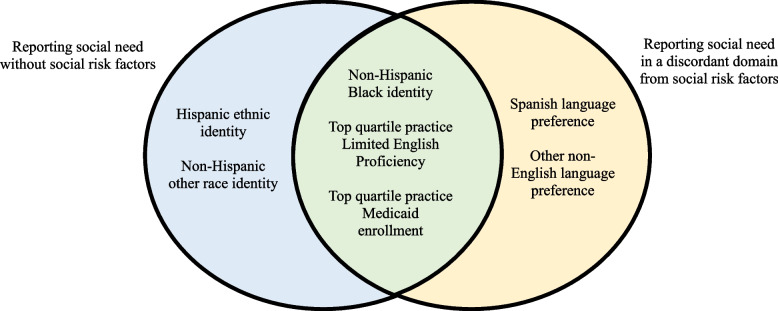
Patient and practice characteristics associated with reporting social needs without social risk factors and/or in a discordant domain from social risk factors

In this study, we demonstrate that screening with social risk factors as compared to social needs identifies different patient populations across a large primary care population in varied practice settings in a Medicaid ACO. Patients who identified as a race/ethnicity other than non-Hispanic White were more likely to report social needs, and more often reported social needs without reporting social risk factors. Among those with social risk factors, patients were more likely to report social needs in a domain discordant to social risks if they identified as non-Hispanic Black, preferred a language other than English, had higher social risk overall, or received care in a practice with higher proportions of patients with LEP and/or Medicaid enrollment. These patients would have been missed if they were screened with social risk factor questions alone (Fig. 1). These individuals are also more likely to experience HRSN due to structural racism and systemic poor access to health services [29, 30], emphasizing the importance of including both social risk factor and social need questions in integrated screening tools to improve the equity and accuracy of clinical screening programs.

It is difficult to precisely compare the prevalence of social risk factors and social needs to other studies due to differences in the populations and screening tools examined. In our study, the 44.6% social risk and 19.1% social need falls in the mid-range of previously published data, with reports of multi-domain HRSN screening in primary care showing a prevalence of 15 to 90% for social risk factors [21, 31, 32], and studies specifically assessing request for assistance finding 15 to 37% with social needs [9, 23, 32]. Our prevalence of food and housing insecurity specifically were also comparable to those reported in other studies [21–23, 31–34].

Our study expands upon prior research identifying a discrepancy between social risk factor and social need screening [23, 25, 35]. This observational study of routine-care screening in a large population across varied practice settings adds to the understanding of HRSN prevalence in clinical practice, and expands upon existing literature by identifying specific patient and practice characteristics associated with domain discordant screening. Our findings are supported by insights from prior research, including a study of an emergency department population in the same health system finding that non-Hispanic Black and Spanish speaking patients more often reported social need rather than social risks [35].

There are multiple reasons why patients may report social needs but not social risk factors on a screening tool. Patients may experience stigma regarding their social circumstances or have privacy concerns about who will see the information [22, 36]. Others may perceive questions on social needs to be more relevant or actionable compared to social risk screening. The finding that patients report social needs in the absence of social risks underscores the limitation of using social risk factor screening alone, and lends further support to implementing patient-centered strategies that engage individuals in determining their own needs and priorities [37].

This study has several potential limitations. First, patients in the sample were only those without substantial churn in Medicaid eligibility (at least 11/13 member-months) and who engaged in routine primary care, limiting the portion of the ACO examined. These patients are likely to be different from the portion of the Medicaid ACO population who experiences more disruptions in eligibility or is unable to participate in scheduled office-based care. Second, the study is a secondary analysis of data that was collected during routine clinical care rather than to answer a specific research question, leading to potential misclassification and missing data. The race, ethnicity, and language data from the EMR were not complete for all included patients, though unavailable data was limited to 8% of race/ethnicity and 3% of language preference. Third, while the institutional screening tool used questions from validated screeners, the entire instrument was not formally validated prior to clinical deployment, leading to potential bias in the patient responses collected. Additionally, we used the request for more information item from this screener to define social need because the answers expressed patient prioritization of a domain and preference for additional engagement. We recognize that patients were not specifically asked if they would like help addressing the health-related social need and this may have led to misclassification of patient responses. The question is an imperfect proxy, although provides an opportunity to understand patient prioritization of their own needs in a real-world clinical screener. Finally, our analysis was limited to patients with Medicaid in a single large, integrated health care system. Because the Medicaid population is more likely to have high social risk and needs, the results may not be generalizable to other patient populations. The practice settings included were varied in size, location, practice ownership, and resources for patients with LEP ranging from on-site interpreters to third-party phone services. However, the results may not be generalizable to patients who are uninsured or who receive medical care in different health system settings.

## Conclusions

The findings from this study have important implications for health policy and practice. Health systems, payors, and policy makers who wish to screen for HRSN should carefully consider how to conduct population-based screening as asking about social risk factors alone are not sufficient to identify all patients with HRSN in a Medicaid population. Populations with systematically higher HRSN may be more likely to report social needs rather than social risk factors. Health systems and Medicaid programs should consider screening tools that include questions which assess both social risk factors and patient-identified social needs. Identifying both populations of patients would increase the opportunity for intervention to reduce the burden of HRSN and associated adverse health outcomes.

## Supplementary Information

**Additional file 1: Supplemental Figure 1.** Health-related social needs institutional screening questionnaire. **Supplemental Table 1.** Characteristics of Medicaid Accountable Care Organization (ACO) patients who completed health-related social needs (HRSN) screening in the payor subset. **Supplemental Table 2.** Social risk factors (positive screening response) and social needs (request for more information) among health-related social needs (HRSN) questionnaires completed in the payor subset. **Supplemental Table 3.** Patient and practice characteristics associated with expressing social need, with and without social risk factors on health-related social need (HRSN) screening in the payor subset. **Supplemental Table 4.** Patient and practice characteristics associated with expressing social need in domains concordant and discordant with social risk factors on health-related social needs (HRSN) screening in the payor subset.

## Data Availability

The datasets created and/or analyzed during the current study are not publicly available due to patient privacy concerns and data confidentiality rules.

## Abbreviations

HRSN: Health-related social needs
ACO: Accountable Care Organization
LEP: Limited English Proficiency
EMR: Electronic Medical Record
GEE: Generalized Estimating Equations
FTE: Full Time Equivalent
IQR: Interquartile Range
