# Supplementary material for: Prevalence of social risk factors and social needs in a Medicaid Accountable Care Organization (ACO)
Source: BMC Health Serv Res. 2022 Nov 19;22:1375. doi: 10.1186/s12913-022-08721-9 (PMC9675191; doi:10.1186/s12913-022-08721-9)
Supplement: Supplementary file 1 — Additional file 1: Supplemental Figure 1. Health-related social needs institutional screening questionnaire. Supplemental Table 1. Characteristics of Medicaid Accountable Care Organization (ACO) patients who completed health-related social needs (HRSN) screening in the payor subset. Supplemental Table 2. Social risk factors (positive screening response) and social needs (request for more information) among health-related social needs (HRSN) questionnaires completed in the payor subset. Supplemental Table 3. Patient and practice characteristics associated with expressing social need, with and without social risk factors on health-related social need (HRSN) screening in the payor subset. Supplemental Table 4. Patient and practice characteristics associated with expressing social need in domains concordant and discordant with social risk factors on health-related social needs (HRSN) screening in the payor subset. [file 12913_2022_8721_MOESM1_ESM.docx]

**Supplemental Figure 1**: Health-related social needs institutional screening questionnaire

**Within the past 12 months we were worried whether our food would run out before we got money to buy more.**

• Never True • Sometimes True • Often True

**Within the past 12 months the food we bought just didn’t last and we didn’t have money to get more.**

• Never True • Sometimes True • Often True

**What is your housing situation today?**

• I do not have housing • I have housing • I choose not to answer

(staying with others, in a hotel,

living outside on the street,

on a bench, in a car, or in a park)

**How many times have you moved in the past 12 months?**

• Three or more times • Two times • One time

• Zero (I did not move) • I choose not to answer

**Are you worried that in the next 2 months, you may not have your own housing to live in?**

• Yes • No • I chose not to answer

**Do you have trouble paying your heating or electricity bill?**

• Yes • No • I chose not to answer

**Do you have trouble paying for medications?**

• Yes • No • I chose not to answer

**Are your currently unemployed and looking for work?**

• Yes • No • I chose not to answer

**Do you have trouble with childcare or the care of a family member?**

• Yes • No • I chose not to answer

**Would you like information today about any of the following topics?**

• Transportation • Food • Housing

• Paying utility bills • Paying for medications • Job search or training

• Education • Childcare • Care for elder or disabled

**In the last 12 months, have you received assistance from an organization or program to help you with any of the following:**

• Transportation • Food • Housing

• Paying utility bills • Paying for medications • Job search or training

• Education • Childcare • Care for elder or disabled

**Supplemental Table 1**: **Characteristics of Medicaid Accountable Care Organization (ACO) patients who completed health-related social needs (HRSN) screening in the payor subset**

| Payor Subset | | |
| --- | --- | --- |
|  | n  (Total = 11093) | % |
| Age |  |  |
| Pediatric (0-18 years) | 5796 | 52.3% |
| Adult (≥ 19 years) | 5297 | 47.7% |
| Sex |  |  |
| Male | 4496 | 40.3% |
| Female | 6624 | 59.7% |
| Race / Ethnicity |  |  |
| Hispanic or Latino | 5261 | 42.0% |
| Non-Hispanic Black or African American | 1580 | 12.6% |
| Non-Hispanic other race | 1325 | 10.6% |
| Non-Hispanic White | 3443 | 27.5% |
| Non-Hispanic unavailable race | 922 | 7.4% |
| Primary Language |  |  |
| English | 7336 | 66.1% |
| Spanish | 2909 | 26.2% |
| Other | 762 | 6.9% |
| Declined | 86 | 0.8% |

**Supplemental Table 2:** **Social risk factors (positive screening response) and social needs (request for more information) among health-related social needs (HRSN) questionnaires completed in the payor subset.**

| Payor Subset | | | | | | |
| --- | --- | --- | --- | --- | --- | --- |
| Social Risk Factors | | | | | | |
|  | n  (Total = 12531) | | | % | | |
| Domains Positive for Risk |  | | |  | | |
| 0 | 6763 | | | 54.0% | | |
| 1 | 3032 | | | 24.2% | | |
| 2 | 1419 | | | 11.3% | | |
| 3+ | 1317 | | | 10.5% | | |
| Positive Domain |  | | |  | | |
| Food insecurity | 2162 | | | 17.3% | | |
| Housing insecurity | 1099 | | | 8.8% | | |
| Medication affordability | 459 | | | 3.7% | | |
| Transportation | 805 | | | 6.4% | | |
| Utilities | 1255 | | | 10.0% | | |
| Child or family care | 550 | | | 4.4% | | |
| Employment | 1504 | | | 12.0% | | |
| Education | 2892 | | | 23.1% | | |
|  | | | | | | |
| Social Needs | | | | | | |
|  | All HRSN Screens | | With Social  Risk Factors | | Without  Social Risk Factors | |
|  | n | % | n | % | n | % |
| Any request | 2884 | 23.0% | 2,389 | 41.4% | 495 | 7.3% |
| Food insecurity | 697 | 5.6% | 610 | 10.6% | 87 | 1.3% |
| Housing insecurity | 984 | 7.9% | 848 | 14.7% | 136 | 2.0% |
| Medication affordability | 232 | 1.9% | 204 | 3.5% | 28 | 0.4% |
| Transportation | 469 | 3.7% | 408 | 7.1% | 61 | 0.9% |
| Utilities | 842 | 6.7% | 1618 | 11.8% | 110 | 1.6% |
| Childcare | 486 | 3.9% | 401 | 7.0% | 85 | 1.3% |
| Care for elder or disabled | 214 | 1.7% | 173 | 3.0% | 41 | 0.6% |
| Job search or training | 673 | 5.4% | 582 | 10.1% | 91 | 1.4% |
| Education | 834 | 6.7% | 736 | 12.8% | 98 | 1.5% |

**Supplemental Table 3**: **Patient and practice characteristics associated with expressing social need, with and without social risk factors on health-related social need (HRSN) screening in the payor subset**

| Payor Subset | | | | | | |
| --- | --- | --- | --- | --- | --- | --- |
|  | Social Need with Social Risk Factors | | | Social Need without Social Risk Factors | | |
|  | PR | CI | P-value | PR | CI | P-value |
| Age |  |  |  |  |  |  |
| Adult (≥ 19 years) | -- | -- |  | -- | -- | -- |
| Pediatric (0-18 years) | 0.9 | 0.9-1.0 | 0.21 | 0.9 | 0.7-1.1 | 0.27 |
| Sex |  |  |  |  |  |  |
| Male | -- | -- |  | -- | -- | -- |
| Female | 1.1 | 1.0-1.2 | 0.04 | 1.1 | 0.9-1.3 | 0.43 |
| Race/ethnicity |  |  |  |  |  |  |
| NH White | -- | -- |  | -- | -- | -- |
| NH Black | 1.3 | 1.2-1.4 | <0.001 | 1.4 | 1.1-1.9 | 0.005 |
| Hispanic | 1.1 | 1.0-1.2 | 0.02 | 1.1 | 0.8-1.4 | 0.46 |
| NH other race | 1.1 | 1.0-1.2 | 0.01 | 1.2 | 0.9-1.5 | 0.31 |
| Primary language |  |  |  |  |  |  |
| English | -- | -- |  | -- | -- | -- |
| Spanish | 1.1 | 1.1-1.2 | <0.001 | 1.2 | 0.8-1.6 | 0.41 |
| Other language | 1.1 | 1.0-1.2 | 0.05 | 1.5 | 1.0-2.3 | 0.04 |
| Number of social risks |  |  |  |  |  |  |
| <3 social risks | -- | -- | -- | -- | -- | -- |
| ≥3 social risks | 2.0 | 1.9-2.1 | <0.001 | -- | -- | -- |
| Practice level social factors |  |  |  |  |  |  |
| 0 practice social factor | -- | -- | -- | -- | -- | -- |
| 1 practice social factor | 1.1 | 1.0-1.3 | 0.008 | 1.2 | 0.8-1.7 | 0.33 |
| 2 practice social factors | 1.1 | 1.0-1.3 | 0.04 | 1.7 | 1.2-2.3 | 0.003 |

Abbreviations: CI, confidence interval; LEP, limited English proficiency; NH, non-Hispanic; PR, prevalence ratio.

Practice level social factors calculated as: 2 practice social factors = highest-quartile LEP and Medicaid; 1 practice social factor = highest-quartile LEP or highest-quartile Medicaid; 0 practice social factor = no highest-quartile practice variable

**Supplemental Table 4:** **Patient and practice characteristics associated with expressing social need in domains concordant and discordant with social risk factors on health-related social needs (HRSN) screening in the payor subset**

| Payor Subset | | | | | | |
| --- | --- | --- | --- | --- | --- | --- |
|  | Social Need in  Concordant Domain | | | Social Need in  Discordant Domain | | |
|  | PR | CI | P-value | PR | CI | P-value |
| Age |  |  |  |  |  |  |
| Adult (≥ 19 years) | -- | -- | -- | -- | -- | -- |
| Pediatric (0-18 years) | 1.0 | 0.9-1.1 | 0.37 | 0.9 | 0.8-1.0 | 0.23 |
| Sex |  |  |  |  |  |  |
| Male | -- | -- | -- | -- | -- | -- |
| Female | 1.1 | 1.0-1.2 | 0.02 | 1.1 | 0.9-1.3 | 0.20 |
| Race/ethnicity |  |  |  |  |  |  |
| NH White | -- | -- | -- | -- | -- | -- |
| NH Black | 1.3 | 1.2-1.5 | <0.001 | 1.2 | 1.0-1.4 | 0.05 |
| Hispanic | 1.2 | 1.1-1.3 | 0.004 | 0.9 | 0.8-1.1 | 0.45 |
| NH other race | 1.2 | 1.1-1.3 | <0.001 | 0.9 | 0.6-1.2 | 0.36 |
| Primary language |  |  |  |  |  |  |
| English | -- | -- | -- | -- | -- | -- |
| Spanish | 1.0 | 0.9-1.1 | 0.83 | 1.7 | 1.5-1.8 | <0.001 |
| Other language | 1.0 | 0.9-1.1 | 0.78 | 1.5 | 1.2-1.9 | <0.001 |
| Number of social risks |  |  |  |  |  |  |
| <3 social risks | -- | -- | -- | -- | -- | -- |
| ≥3 social risks | 2.9 | 2.7-3.1 | <0.001 | 0.5 | 0.4-0.7 | <0.001 |
| Practice level social factors |  |  |  |  |  |  |
| 0 practice social factor | -- | -- | -- | -- | -- | -- |
| 1 practice social factor | 1.2 | 1.0-1.3 | 0.03 | 1.1 | 0.9-1.3 | 0.37 |
| 2 practice social factor | 1.1 | 1.0-1.2 | 0.10 | 1.3 | 1.0-1.5 | 0.02 |

Abbreviations: CI, confidence interval; LEP, limited English proficiency; NH, non-Hispanic; PR, prevalence ratio.

Practice level social factors calculated as: 2 practice social factors = highest-quartile LEP and Medicaid; 1 practice social factor = highest-quartile LEP or highest-quartile Medicaid; 0 practice social factor = no highest-quartile practice variable
